# The provision of non-needle/syringe drug injecting paraphernalia in the primary prevention of HCV among IDU: a systematic review

**DOI:** 10.1186/1471-2458-10-721

**Published:** 2010-11-23

**Authors:** Michelle Gillies, Norah Palmateer, Sharon Hutchinson, Syed Ahmed, Avril Taylor, David Goldberg

**Affiliations:** 1 Department of Public Health-Faculty of Medicine, University of Glasgow, G12 8QR, UK; 2 BBV and STI Section, Health Protection Scotland, Clifton House, Clifton Place, Glasgow, G3 7LN, UK; 3 Department of Mathematics and Statistics, University of Strathclyde, Livingstone Tower, 26 Richmond Street, Glasgow, G1 1XH, UK; 4 Public Health Protection Unit, NHS Greater Glasgow & Clyde, Dalian House, 350 St Vincents Street, Glasgow, G3 8YU, UK; 5 Institute for Applied Social and Health Research, University of the West of Scotland, Paisley, PA1 2BE, UK

## Abstract

**Background:**

Sharing drug injecting paraphernalia other than needles and syringes (N/S) has been implicated in the transmission of Hepatitis C virus (HCV) among injecting drug users (IDU). We aimed to determine whether the provision of sterile non-N/S injecting paraphernalia reduces injecting risk behaviours or HCV transmission among IDU.

**Methods:**

A systematic search of seven databases and the grey literature for articles published January 1989-February 2010 was undertaken. Thirteen studies (twelve observational and one non-randomized uncontrolled pilot intervention) were identified and appraised for study design and quality by two investigators.

**Results:**

No studies examined the association between the provision of non-N/S injecting paraphernalia and incident HCV infection. One cross-sectional study found that individuals who frequently, compared to those who infrequently, used sterile cookers and water, were less likely to report prevalent HCV infection. Another found no association between the uptake of sterile non-N/S injecting paraphernalia and self-reported sharing of this paraphernalia. The remaining observational studies used attendance at needle and syringe exchange programmes (NSP) or safer injection facilities (SIF) that provided non-N/S injecting paraphernalia as a proxy measure. Eight studies presented adjusted odds ratios, ranging from 0.3 to 0.9, suggesting a reduced likelihood of self-reported sharing of non-N/S injecting paraphernalia associated with use of NSP or SIF. There was substantial uncertainty associated with these estimates however. Three unadjusted studies reported a reduction in the prevalence of sharing of non-N/S injecting paraphernalia over time among NSP users. Only one study reported an adjusted temporal trend in the prevalence of sharing non-N/S injecting paraphernalia, finding higher rates among non-NSP users than NSP users at each time point, and a greater reduction in sharing among non-NSP than NSP users over time. Study limitations included the use of convenience samples, self-reported exposure and outcome measures, flawed classification of the exposed and unexposed groups, and inadequate adjustment for potential confounding variables.

**Conclusions:**

The evidence to demonstrate that the provision of sterile non-N/S injecting paraphernalia reduces HCV transmission or modifies injecting risk behaviours is currently limited by an insufficient volume and quality of studies. Further research is required to inform practice and policy in this area.

## Background

The sharing of drug injecting paraphernalia, equipment used in the preparation and administration of drugs for injection, is common and has been implicated in the transmission of Hepatitis C (HCV) [1-3]. There is compelling evidence that HCV transmission occurs through the sharing of contaminated needles and syringes (N/S). This is biologically plausible; in the act of preparing and administering drugs for injection, N/S will generally come into direct contact with blood. The prevalence of HCV is high among those who report injecting drug use [4] and the risk of HCV transmission has been independently documented in studies of needle-stick injury in health care workers [5,6].

Evidence relating to the risk of HCV transmission associated with sharing non-N/S injecting paraphernalia is less well established however. Ethnographic studies have highlighted numerous opportunities for cross-contamination to occur when individuals share drug preparation equipment other than N/S [7-10]. Laboratory studies have isolated HCV RNA from injecting equipment including spoons used as drug cookers (also called stericups), filters (also called cottons or sterifilts) and water samples [11]. The self-reported sharing of such items of paraphernalia is high, irrespective of the setting or population studied. Between 65% to 84%, 50% to 77%, and 15% to 83% of intravenous drug users (IDU) report sharing drug cookers, filters and water, respectively [12].

To date, few longitudinal observational studies have detected an association between HCV incidence and the sharing of non- N/S injecting paraphernalia. A recent systematic review concluded that evidence relating the sharing of such drug injecting paraphernalia to HCV transmission was 'not overwhelming' and highlighted several methodological limitations of the studies that contribute to this evidence base [13]. A high baseline prevalence of HCV among IDU and poor study retention has limited the statistical power that many studies have had to detect an association between incident HCV infection and isolated, often highly correlated, injecting practices.

In the United Kingdom (UK) primary and secondary health care is provided, free at point of access, by the National Health Service (NHS). Local authorities are responsible for meeting nationally agreed targets on health and social care but retain considerable autonomy over the allocation of resources. Following legislative change in 2003, N/S exchange programmes (NSP) in the UK are now able to provide clients with sterile items of drug injecting paraphernalia in addition to N/S. There is however wide local and regional variation in the provision of such paraphernalia by NSP [14,15]. The effectiveness, and indeed cost-effectiveness, of providing of sterile injecting paraphernalia other than needles and syringes in the primary prevention of HCV among IDU has yet to be established [16]. To inform future public health policy and practice in the UK and elsewhere, we conducted a systematic review to determine whether the distribution of sterile drug injecting paraphernalia other than needles and syringes reduces the risk of HCV transmission or modifies injecting risk behaviours.

## Methods

We searched MEDLINE, MEDLINE In- Process & Other Non-Indexed Citations, Cochrane Central Register of Controlled Trials, Cochrane Database of Systematic Reviews, Database of Abstracts of Reviews of Effects, EMBASE and PsycINFO for articles published between January 1989 and February 2010 using the search strategy outlined in Appendix 1. The grey literature was searched using the terms 'Hepatitis C' or 'HCV' and 'paraphernalia'. Reference lists of selected articles were reviewed and citation checks carried out to identify further potentially relevant studies. Two investigators (MG, NP) reviewed the abstracts of potentially eligible articles to determine relevance; where relevant full texts were retrieved and examined.

### Inclusion and exclusion criteria

Injecting paraphernalia was defined as equipment used in the preparation or administration of drugs for injection. For the purposes of this review we limited this definition to drug cookers, filters and water, as these are the items of injecting paraphernalia most likely to be contaminated with blood in the course of preparing or administering drugs [7-10]. The exposure of interest was the distribution of drug cookers, filters and/or water; however, the self-reported uptake of these items or the use of an NSP or safer injection facility (SIF) that provided these items was accepted as a proxy measure of exposure. Studies that did not provide one or more of these items of paraphernalia or that did not explicitly state which items of paraphernalia were provided, were excluded. The outcomes of interest were (i) incident HCV infection (ii) prevalent HCV infection and (iii) injecting risk behaviours, namely the self-reported sharing of drug cookers, filters and/or water. The study population of interested was current IDU. Descriptive studies, qualitative studies, review articles, editorials and opinion pieces were excluded. Only primary research studies were considered. Where two or more articles presented data from the same cohort during overlapping time frames, the publication with the largest sample size was selected for inclusion.

### Study Appraisal

We used the STROBE [17] criteria as a guide to consider key aspects of study design: sampling methods, definition of study population, inclusion and exclusion criteria, assessment of exposure, assessment of outcome(s), completeness of follow-up, treatment of missing data and treatment of possible confounding variables. Each item was assessed in terms of how well the area was addressed and reported by investigators in relation to the aims of this review. Study quality was assessed independently by two reviewers (MG, NP) with disagreement resolved by a third reviewer (SH).

## Results

**Figure 1:**
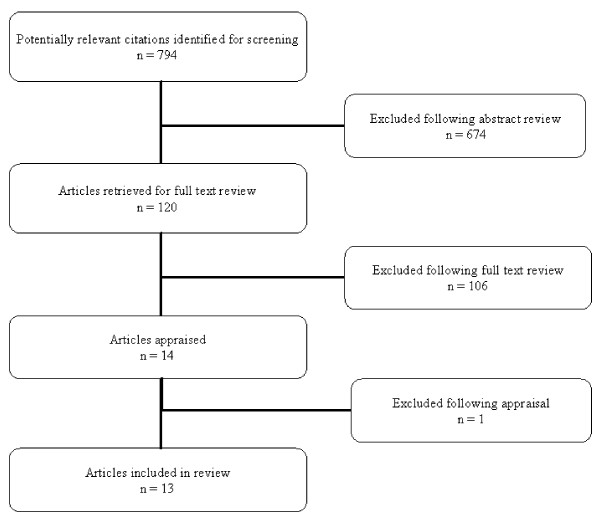
**Inclusion and exclusion of studies through the review process**.

From the 794 citations retrieved fourteen studies met the inclusion criteria (Figure 1). Following appraisal, one unpublished cross-sectional study was rejected [18]. The remaining studies - five cohort studies [19-23], one serial cross-sectional study [24], six cross-sectional studies [25-30] and one non-randomised intervention study [31] - were conducted in North America (11 in the USA [19-21,23,24,26-31] and two in Canada [22,25]) between 1992 and 2005 (Table 1). With one exception [30], all examined the association between use of an NSP (including secondary exchange of N/S [28]) or SIF [22] at which sterile non- N/S injecting paraphernalia were available, and the self-reported sharing of this equipment. We did not identify any studies reporting the risk of incident HCV infection in relation to the use of sterile injecting paraphernalia; however, one cross-sectional study reported the likelihood of using sterile drug cookers and sterile water according to self-reported HCV status [25].

**Table 1:** Summary of studies included in review following appraisal

Reference	Design	Setting	Study Population	Data Collected	Study Limitations
Hagan *et al*. 2000 [19]	Cohort	Seattle, USA Recruitment: 1994 - 1997	Random sample of IDU recruited from locations other than NSP including prisons, street outreach, social services and drug treatment programmes. 2,814 IDU aged 14 years or older who had injected elicit drugs in the last year of whom 2,208 (78.5%) completed a follow-up interview. NSP users (n = 1236) had 'ever' used the NSP in the period prior to enrolment; non-users (n = 346) had 'never' used NSP prior to. IDU paid to participate. The NSP provided drug cookers, cottons and rinse water.	Exposure: NSP use ('Ever' *vs*. 'Never') defined at study enrolment. Outcome: Self-reported sharing of 'cookers or cottons' in the month and six months prior to study enrolment. Thereafter behaviours in the month prior to follow up. Data collected by interview administered questionnaire at baseline and follow up visit at 1 year.	Dichotomous measure of NSP use 'ever' *vs*. 'never' does not allow for gradations in use. NSP use at baseline may not represent NSP use at follow up. Self-reported outcome measures. Statistical analyses were carried out on 71.6% (n = 1,582) participants who completed a follow up visit, representing 56.2% of all eligible participants enrolled at baseline. The authors report no association between study retention and NSP use or injecting risk behaviour, however high loss to follow up may have reduced statistical power.
Huo *et al *2007 [20]	Cohort	Chicago, USA Recruitment: 1997 - 2000	Convenience sample of IDU that had injected in the last 6 months and were aged 18 years or older. Verification of age and recent IDU required. Regular NSP users (n = 729) recruited at NSP (multisite). Non NSP users (n = 172), individuals who had not used a NSP in the 6 months prior to study enrolment, were recruited from a 'control' area without local NSP services via street outreach and peer referral. The NSP supplied cotton, cookers, alcohol wipes and water. Non-users had access to cookers.	Exposure: NSP use (Regular *vs*. non NSP users) defined at study enrolment. Outcome: Self-reported prevalence of sharing 'other' injecting paraphernalia in the 30 days prior to interview. This composite endpoint was used to denote the sharing of any single item of non-N/S paraphernalia. Data collected by interview administered questionnaire at baseline and three annual follow up visits.	Convenience sampling Differential sampling method NSP users and non-users. Dichotomous measure of NSP use does not allow for gradations in use. NSP use defined at baseline may not represent NSP use at follow up. Both NSP users and non-users had access to sterile injecting paraphernalia thereby limiting comparison between groups. Self-reported outcome measures Follow up data available on 83.7% (n = 610) NSP users and 75.6% (n = 130) non NSP users (did not differ according to risk behaviour at baseline).
Sears *et al*. 2001 [21]	Cohort	San Francisco, USA. Recruitment: July 1993	NSP users recruited using systematic sampling (every *n*th person) of IDU attending NSP in three locations. Non-users were recruited using a targeted sampling method through an ongoing multi-wave cross-sectional behavioural study that included HIV testing and counselling (the Urban Health Study). Participation rate for NSP users 88%, for non-users 84%. NSP status subsequently reclassified according to reported NSP use at baseline rather than recruitment site. NSP users (n = 132) reported at least one use of NSP in the last 30 days and at least one additional use of NSP in the preceding 6 months. Non-users (n = 97) reported no use of NSP in the past 30 days or the preceding 6 months. IDU paid to participate. The NSP provided cottons and alcohol swabs.	Exposure: NSP use (NSP use *vs*. non use) defined at study enrolment. Outcome: Self-reported sharing of cotton, cooker or water in the preceding 30 days. Data collected during structured interviews at baseline, 6 month and 12 month follow up.	Differential sampling methods in recruitment of NSP users and non-users (possible selection bias). Although the outcome measure was sharing of cottons, cookers or water, participants only had access to cottons and alcohol swabs from NSP. Dichotomous measure of NSP use excludes gradations in use. NSP use at baseline may not reflect NSP use at follow-up. Self-reported outcome measures. Follow up data collected on 49% and 57% of participants at 6 and 12 month visits respectively. Differential follow up according to NSP use; in the non-users group those lost to follow up reported higher risk injecting behaviours. Bivariate analyses carried out on complete cases only (n = 101, 44% of participants). Data from full sample included in multivariate analysis taking into account random missing data. AOR stratified by NSP status not reported. P-values not provided for bivariate analysis of reported prevalence of sharing 'cooker, cotton, or water' according to NSP use over time.
Stoltz *et al*. 2007 [22]	Cohort	Vancouver, Canada. Recruitment: 2004 - 2005	Random sample of IDU attending a medically supervised injection facility (SIF). 760 IDU who completed a follow up survey between 1^st ^July 2004 and 30^th ^June 2005. Frequent SIF users (n = 433) were defined as those who used the SIF for some, most or all of their injecting episodes (i.e. > 25% of all injecting episodes) in the month prior to interview. Infrequent SIF users (n = 327) used the facility for < 25% of injecting episodes. SIF use determined at baseline. Participation rate not reported. Facility provided a location to inject drugs intravenously under the supervision of a nurse, advice on safe injecting practices, referral to health care and addiction services and medical intervention in the event of an overdose. IDU paid to participate. Paraphernalia provided included alcohol swabs, sterile water and cookers.	Exposure: SIF use (frequent *vs*. infrequent) defined at study enrolment. Outcome: Self-reported change in using clean water for injecting since using the SIF. Data collected by interview administered questionnaire at baseline and 6 month follow-up.	Baseline classification of SIF use may not represent SIF use at follow up. Dichotomous measure of SIF use does not allow for gradations in use. Losses to follow up not documented although authors report no difference in the baseline characteristics of the cohort according to follow up status. Self-reported outcome measures.
Vlahov *et al*. 1997 [23]	Cohort	Baltimore, USA. Recruitment: 1994 - 1995	Systematic sampling of all self-reported IDU enrolled in the NSP during the first year of operation. 422 IDU completed baseline interviews (14.2% of all individuals enrolled in the NSP). IDU paid to participate. NSP provided injection kits containing alcohol wipes, cotton and cookers.	Exposure: NSP use (before and after establishment of NSP). Outcome: Self-reported sharing of cookers or cottons in the preceding 2 weeks. Data collected by interview administered questionnaire at baseline (NSP inception), 2 weeks and 6 months	Self-reported outcome measures. Unadjusted analyses only. No comparison group. Follow up 79.4% (n = 335) at 2 weeks, 52.4% (n = 221) at 6 months. Individuals lost to follow up differed with respect to injecting risk behaviours from those with complete data.
Bluthenthal *et al*. 1998 [24]	Serial Cross- Sectional Studies	California, USA. Recruitment: 1992 - 1995	Targeted sample of IDU aged 18 years or older reporting injecting drug use in the preceding 30 days, recruited by street outreach and snowballing techniques. Verification of recent IDU required. Individuals who participated were encouraged to return on subsequent semi-annual waves. Total of 1034 IDU interviewed once with 53% (n = 684) returning for one or more follow up visit. Setting: Illegal NSP. NSP users had used the NSP in the 30 days prior to interview to obtain injecting equipment and NSP was the usual source of syringes in the 6 months prior to interview. NSP provided alcohol and cotton filters. Individuals who had received other risk reduction supplies such as cottons and cookers from any HIV provider in the 30 days prior to interview were identified.	Exposure: NSP use (NSP users *vs*. non-users and trends over time). Outcome: Self-reported sharing of 'cookers, cottons or rinse water' in the 30 days prior to interview. A composite outcome of sharing injection supplies was used to indicate the self-reported sharing of any single item of non- N/S paraphernalia. Data collected by interview administered questionnaire in seven semi-annual waves.	Participants only had access to cottons and alcohol wipes from NSP thereby limiting the ability to interpret results. Convenience sampling. Self-reported outcome measures.
Morissette *et al*. 2007 [25]	Cross-sectional	Montreal, Canada. Recruitment: 2004 - 2005	Convenience sample of IDU (n = 275) aged 18 years or older who had injected at least once in the last 6 months recruited by flyers, peer referral and onsite personnel at three large NSP. Verification of recent injecting required. Participation rate not reported. IDU paid to participate. NSP distributed sterile water and Securicup kits containing cooker, filter and swabs.	Exposure: Use of sterile non-N/S injecting equipment (frequent vs. infrequent). Outcome: Self-reported sharing of drug preparation equipment (cookers, filters, water) in the preceding 6 months (yes/no), and frequency of use of sterile cookers, filters and water in the preceding month, HCV status. Source of sterile injecting equipment and reasons for not using sterile equipment in the past 6 months. Data collected by interview-administered questionnaire.	Convenience sample. Self-reported outcome measures.
Longshore *et al*. 2001 [26]	Cross-sectional	Rhode Island, USA. Recruitment: 1997 - 1998	Convenience sample of IDU over the age of 18 yrs old attending NSP or MMT. 248 IDU (participation rate 70%). IDU paid to participate. NSP supplied cotton, cookers, alcohol wipes and rinse water.	Exposure: NSP use (frequent *vs*. infrequent use). Outcome: Self-reported sharing of cooker or other items of injecting paraphernalia in the 6 months prior to interview and frequency of visits to NSP. Responses treated as categorical variable. Data collected by interview administered questionnaire.	Convenience sample. Self-reported outcome measures. No comparison group.
Kipke *et al*. 1997 [27]	Cross-sectional	California, USA. Recruitment: 1994	Convenience sample of IDU aged 16-24 years who had injected at least once during the last 30 days. 89 NSP users recruited through NSP (mobile van). 109 non-users (poorly characterised) recruited by street outreach and using snowballing techniques. Participation rate not provided. IDU paid to participate. NSP provided water, cotton, cookers, alcohol wipes.	Exposure: NSP use (NSP users *vs*. non-users). Outcome: Self-reported sharing of 'cookers, cottons or rinse water' in the preceding 6 months. Data collected during semi-structured interview.	Convenience sample. Differential sampling methods between NSP users and non NSP users. Flawed assessment of exposure to NSP and access to paraphernalia. NSP non-users poorly characterised. Self-reported outcome measures.
Sears *et al*. 2001 [28]	Cross-sectional	San Francisco, USA. Recruitment: July - Sept. 1997	Convenience sample of IDU aged 15-25 years who had injected drugs in the last 30 days and had experienced homelessness within the last 30 days. Recruitment in two areas of city: one in which a secondary exchange programme operated (intervention site) and a comparison site near which two NSP operated. 67 IDU recruited from the intervention site and 55 from the comparison site (100% participation). IDU paid to participate. Secondary syringe exchange programme led by 4 peer exchangers provided cookers, filters, water and alcohol swabs. Items of paraphernalia provided by other NSP unclear.	Exposure: Access to secondary syringe exchange *vs*. access to other NSP (comparison based site of recruitment). Outcome: Self-reported sharing of cookers in the preceding 30 days. Data collected during structured interview.	Convenience sample. Almost 90% of study population used NSP. In the comparison group almost 40% reported obtaining sterile paraphernalia from secondary exchange, half from a recognised formal NSP, a quarter from an underground NSP and three quarters via satellite distribution. Self-reported outcome measures. Results of adjusted analysis not presented.
Heimer *et al*. 2002 [29]	Cross- sectional	Multi-site study Connecticut, California, Illinois, USA. Recruitment: 1998 - 2000	Targeted sample of IDU aged 18 years or older reporting injecting drug use in the preceding 30 days, recruited by street outreach and modified snowballing. 493 IDU (n = 122 NSP users, n = 371 NSP nonusers). Participation rate not provided. NSP provided water but unclear whether other items of injecting paraphernalia such as cookers or filter were provided.	Exposure: NSP use (NSP users *vs*. non-users). Outcome: Self-reported sharing of cookers, cottons, rinse water or drug water in the 30 days prior to interview. In addition information on HIV and hepatitis knowledge was collected. Data collected during semi-structured interview	NSP users and nonusers not clearly defined. Paraphernalia provided by NSP poorly defined. Convenience sampling. Self-reported outcome measures. Cohort study but cross-sectional data only presented. Unadjusted analyses comparing sharing of injecting paraphernalia among NSP users and nonusers only.
Guydish *et al*. 1998 [30]	Cross-sectional	San Francisco, USA. Recruitment: October 1994	Systematic sampling of all self-reported IDU attending multi-site NSP. Study population poorly characterised. 143 IDU recruited of whom 114 (80%) participated. IDU paid to participate. NSP distributed cotton and alcohol wipes.	Exposure: NSP use Outcome: Self-reported sharing of rinse water in the 30 days prior to interview. The sharing rinse water selected to represent all 'indirect' sharing. Also collected information on the proportion of all needles used by a client that came from the NSP and frequency of visits to NSP in last 30 days. Data collected by interview administered questionnaire.	Rinse water was selected to represent all 'indirect' sharing; NSP did not distribute rinse water thereby limiting the ability to interpret results. Study population poorly characterised. Self-reported outcome measures. Only unadjusted analyses presented. No comparison group.
Colon *et al*. 2009 [31]	Non-randomised intervention study (pilot)	Puerto Rico, USA. Recruitment: 16 week intervention period of recruitment not described	Targeted sample of IDU aged 18 years or older reporting injecting drug use in the preceding 7 days recruited by street outreach. 37 IDU (70% participation). 16 week community level intervention aimed at modifying injecting risk behaviours. IDU provided with injection kits that included hand sanitizer, sterile water in a water bottle and syringe filter and advised on use.	Exposure: Community level intervention (see study population) Outcome: Self-reported change in injecting practices in the preceding 7 days: use of hand sanitizer, use of water bottle and use of syringe filter provided in intervention, use of non-study materials including water and cookers. Self-reported sharing of cooker during last day of injection. Data collected by interview administered questionnaire. Pre and post intervention data collected by structured interview. Analysis of systematically sampled water bottles and cookers for the presence of red blood cells. Samples collected from shooting galleries 4 and 1 weeks prior to intervention and 8, 14 and 18 weeks following commencement of the intervention.	Non-randomised, uncontrolled study design Convenience sample. Pilot study with small numbers therefore unable to generalise findings to other populations.

### Study Quality

The major methodological limitations identified include the use of convenience samples [20,25-28], self-reported exposure and outcome measures [19-30], the use of NSP (or SIF) as a proxy measure for use of sterile non- N/S injecting equipment [19-24,26-31], in some cases with inadequate assessment of exposure to NSP [20,27,28] and statistical analyses which failed to adjust for potential confounding variables [23,24,27,29,30] (Table 1). None of the cohort studies identified undertook traditional cohort analyses [19-23]. Participation rates (reported in 5 studies) varied from 70% to 100% and losses to follow up (reported in 4 of the 5 cohort studies) ranged from 27% to 56%. The only non-observational study was a small (n = 37) uncontrolled non-randomized pilot intervention trial [31].

### Cohort Studies

Five cohort studies examining over 5,000 IDU between 1994 and 2005 were included in this review [19-23] (Table 2). The largest study followed 2,814 IDU, recruited in Seattle, USA from 1994 through 1997, over a one year period [19]. The authors found no significant difference in the sharing of 'cookers or cottons' in the month prior to follow-up interview between those who reported 'ever' compared to 'never' using an NSP at study enrolment, with an adjusted odds ratio (AOR) of 0.79 (95% CI 0.58 - 1.08). Huo *et al *documented a high prevalence of sharing 'other injecting paraphernalia' in the 30 days prior to interview in both NSP users (60%) and nonusers (70%) at baseline in their cohort of 901 IDU recruited in Chicago, USA between 1997 and 2000 [20]. Across the 3 year study the self-reported prevalence of sharing 'other injecting paraphernalia' fell to almost identical levels in both NSP users and nonusers (approximately 22%). Following adjustment, NSP users were less likely, across all study visits, to report sharing 'other injecting paraphernalia' in the preceding month compared to non-users (AOR 0.7, (0.52 - 0.95)). Sears and colleagues compared the self-reported sharing of 'cottons, cookers or water' in the 30 days prior to interview at baseline, 6 months and 1 year follow up in NSP users (n = 132) and non-users (n = 97) recruited in San Francisco, USA in 1993 [21]. Due to differential attrition rates according to NSP status, bivariate analyses were limited to cases that completed all three interviews (n = 101, 44% of the study population). NSP users were "significantly" more likely than non-users to report sharing a 'cotton, cooker or water' at baseline (61% NSP users *vs*. 40% non-users, p value not reported). A decline in the prevalence of sharing a 'cotton, cooker or water' at follow up was reported in both NSP users (to approximately 55% at 6 months and 45% at 1 year) and nonusers (to approximately 40% at 6 months and 30% at 1 year); p-values were not provided. In multivariate analyses there was an interaction between NSP status by study visit for the sharing of a 'cotton, cooker or water' was reported. AOR stratified by NSP status were not presented, however the authors concluded "nonexchangers showed a larger decrease than exchangers in this behaviour over time." More contemporaneous data (2004 - 2005) from Vancouver, Canada explored changes in injection practices associated with use of a medically supervised safer injection facility (SIF) [22]. Consistent SIF users (n = 433) - individuals that reported using the SIF for greater than 25% of all injecting episodes - were more likely than inconsistent users (n = 327) to report a change to using clean water for injecting since they started using the SIF (AOR 2.99 (2.13 - 4.18)). Lastly, Vlahov and colleagues carried out a before -after study following a cohort of 422 IDU from enrolment in a newly established NSP in Baltimore, USA over a six month period [23]. The authors observed a significant reduction in the proportion of participants that reported sharing cotton filters in the two weeks prior to interview, from 46% at baseline to 31% at six month follow up visit (P < 0.001). This trend was also observed for cooker sharing, for which the corresponding values were 59% and 39%, respectively (P < 0.001). Adjusted analyses were not presented.

**Table 2:** Summary of main research findings of studies included in review

Reference	Paraphernalia	Exposure	Outcome Measure	Measure of Association
Hagan *et al*. 2000 [19]^a^	Cooker or Filter	NSP users *vs*. nonusers	AOR (95%CI) of sharing paraphernalia with adjustment for heroin injection, frequency of injection, and syringe or cooker/cotton sharing or back loading	0.79 (0.58 - 1.08)
Huo *et al*. 2007 [20]^a^	Cooker, Filter or rinse water	NSP users *vs*. nonusers	Proportion of participants reporting sharing paraphernalia (behaviour at baseline to behaviour at 3 year follow up)	NSP users: 60% to ~22% NSP nonusers: 70% to ~22%
Huo *et al*. 2007 [20]^a^	Cooker, Filter or rinse water	NSP users *vs*. nonusers	AOR (95%CI) of sharing paraphernalia with adjustment for year recruited, age, race, heroin or cocaine injection, injecting with others at least half the time, injecting in semi-public settings, injecting in shooting gallery, and having an IDU sex partner	0.70 (0.52 - 0.95)
Sears *et al*. 2001 [21]^a^	Cooker, Filter or rinse water	NSP users *vs*. nonusers (complete case analysis, n = 101)	Proportion of participants reporting sharing paraphernalia (behaviour at baseline to behaviour at 1 year follow up)	NSP users: 61% to 45% NSP nonusers: 40% to 30%
Stoltz *et al*. 2007. [22]^a^	Water	Frequent SIF user *vs*. infrequent SIF user	AOR (95%CI) of using clean water for injecting with adjustment for age, gender, sex trade involvement, daily cocaine and heroin injection	2.99 (2.13 - 4.18)
Vlahov *et al*. 1997 [23]^a^	Cooker	Pre- vs. post-enrolment in NSP	Proportion of participants reporting sharing paraphernalia (at baseline and at 6 months)	59% to 39% ^d^
Vlahov *et al*. 1997 [23]^a^	Filter	Pre- vs. post-enrolment in NSP	Proportion of participants reporting sharing paraphernalia (at baseline and at 6 months)	46% to 31% ^d^
Bluthenthal *et al*. 1998 [24]	Cooker, Filter or rinse water	Temporal trends in prevalence of sharing paraphernalia	Proportion of participants reporting sharing paraphernalia between wave 1 (1992) and wave 7 (1995)	~62% to ~40% ^d^
Bluthenthal *et al*. 1998 [24]	Cooker, Filter or rinse water	Receipt of sterile injecting paraphernalia in last 30 days *vs*. not	AOR (95%CI) of sharing paraphernalia with adjustment for wave of study recruitment, gender, neighbourhood, homelessness, HIV status, race, age, frequency of injection and drug preference.	1.11 (0.89 - 1.39)
Bluthenthal *et al*. 1998 [24]	Cooker, Filter or rinse water	NSP users *vs*. nonusers	AOR (95%CI) of sharing paraphernalia with adjustment for wave of study recruitment, gender, neighbourhood, homelessness, HIV status, race, age, frequency of injection and drug preference.	0.85 (0.68 - 1.07)
Morissette *et al*. 2009 [25]	Cooker	Use of sterile injecting equipment 'half the time or more' *vs*. 'less than half the time' in the last month	OR (95%CI) of sharing drug preparation equipment in the last 6 months	0.42 (0.22 - 0.80)
Morissette *et al*. 2009 [25]	Filter	Use of sterile injecting equipment 'half the time or more' *vs*. 'less than half the time' in the last month	OR (95%CI) of sharing drug preparation equipment in the last 6 months	0.52 (0.28 - 0.97)
Morissette *et al*. 2009 [25]	Water	Use of sterile injecting equipment 'half the time or more' *vs*. 'less than half the time' in the last month	OR (95%CI) of sharing drug preparation equipment in the last 6 months	0.51 (0.29 - 0.90)
Morissette *et al*. 2009 [25]	Cooker	Use of sterile injecting equipment 'half the time or more' *vs*. 'less than half the time' in the last month	AOR (95%CI) of being HCV negative *vs*. HCV positive (self-reported) adjusted for age, gender, education, injecting heroin as a primary drug, daily injecting, injecting alone, requiring help with injecting and problems obtaining sterile equipment from a NSP	3.92 (1.58 - 9.70)
Morissette *et al*. 2009 [25]	Filter	Use of sterile injecting equipment 'half the time or more' *vs*. 'less than half the time' in the last month	AOR (95%CI) of being HCV negative *vs*. HCV positive (self-reported) adjusted for age, gender, education, injecting heroin as a primary drug, daily injecting, injecting alone, requiring help with injecting and problems obtaining sterile equipment from a NSP	No association ^g^
Morissette *et al*. 2009 [25]	Water	Use of sterile injecting equipment 'half the time or more' *vs*. 'less than half the time' in the last month	AOR (95%CI) of being HCV negative *vs*. HCV positive (self-reported) adjusted for age, gender, education, injecting heroin as a primary drug, daily injecting, injecting alone, requiring help with injecting and problems obtaining sterile equipment from a NSP	2.93 (1.12 - 7.68)
Longshore *et al*. 2001^e ^[26]	Cooker	NSP attendance > 4 times *vs*. once or less/month	AOR (95%CI) of sharing paraphernalia with adjustment for age, race, sex, injection frequency, primary drug, and treatment experience.	0.39 (0.16 - 0.95)^c^
Longshore *et al*. 2001^e ^[26]	Cooker	NSP attendance > 4 times *vs*. 2-4 times/month	AOR (95%CI) of sharing paraphernalia with adjustment for age, race, sex, injection frequency, primary drug, and treatment experience.	0.50 (0.28 - 0.89) ^c^
Longshore *et al*. 2001^e ^[26]	Filter	NSP attendance > 4 times *vs*. once or less/month	AOR (95%CI) of sharing paraphernalia with adjustment for age, race, sex, injection frequency, primary drug, and treatment experience.	0.64 (0.27 - 1.52)
Longshore *et al*. 2001^e ^[26]	Filter	NSP attendance > 4 times *vs*. 2-4 times/month	AOR (95%CI) of sharing paraphernalia with adjustment for age, race, sex, injection frequency, primary drug, and treatment experience.	0.88 (0.49 - 1.59)
Kipke *et al*. 1997 [27]	Cooker, Filter or rinse water	NSP users *vs*. nonusers	AOR (95%CI) of sharing paraphernalia with adjustment for age, gender and ethnicity	0.53 (0.28 - 0.99)^c^
Sears *et al*. 2001 [28]	Filter	Intervention site *vs*. comparison site^f^	AOR (95%CI) of using someone else's cotton with adjustment for age, race/ethnicity, illegal/marginal income source, use of drop-in centre, and number of times consumed alcohol in the past 30 days	No association ^g^
Heimer *et al*. 2002. [29]	Cooker	NSP users *vs*. nonusers	Proportion of participants reporting sharing paraphernalia in last 30 days	9% *vs*.12% ^b^
Heimer *et al*. 2002. [29]	Filter	NSP users *vs*. nonusers	Proportion of participants reporting sharing paraphernalia in last 30 days	6% *vs*.8% ^b^
Heimer *et al*. 2002. [29]	Rinse water	NSP users *vs*. nonusers	Proportion of participants reporting sharing paraphernalia in last 30 days	3% *vs*. 9% ^d^
Heimer *et al*. 2002. [29]	Drug water	NSP users *vs*. nonusers	Proportion of participants reporting sharing paraphernalia in last 30 days	3% *vs*.9% ^c^
Guydish *et al*. 2008 [30]	Cooker, Filter or rinse water	NSP users who reported sharing rinse water in preceding 30 days *vs*. those who did not	Mean NSP visits in past 30 days	4.36 *vs*. 3.75 ^b^
Guydish *et al*. 2008 [30]	Cooker, Filter or rinse water	NSP users who reported sharing rinse water in preceding 30 days *vs*. those who did not	Mean percent of syringes obtained from NSP	86% *vs*. 89% ^c^
Colon *et al*. 2009 [31]	Water bottle	Pre *vs*. post intervention (18 week follow up)	Percentage of participants adopting injecting practices in the preceding 7 days	0% *vs *56%^d,h^
Colon *et al*. 2009 [31]	Syringe filter	Pre *vs*. post intervention (18 week follow up)	Percentage of participants adopting injecting practices in the preceding 7 days	0% *vs*. 34%^c,h^
Colon *et al*. 2009 [31]	Cooker	Pre *vs*. post intervention (18 week follow up)	Percentage sharing cooker during last day of injection	16% *vs*. 6% ^b^

^a^cohort studies did not report risk ratios: see Results section for details, ^b^not significant, ^c^P < 0.05, ^d^P < 0.001, ^e^odds ratios presented here use the group with lower frequency of NSP use as the referent group (as compared to those presented in the text), ^f^intervention site were recruited from an area in which a secondary exchange operated, comparison site were recruited from another location near which two other NSP operated, ^g ^result not presented, ^h^practices absent at baseline therefore p values for test against null hypothesis that proportions at follow up < 15%

### Serial Cross-sectional Studies

Bluthenthal *et al *conducted serial cross-sectional studies in seven semi-annual waves over a three year period in two neighbourhoods in California, USA in which an illegal NSP distributed injecting kits containing alcohol wipes and cotton filters [24]. Between the first and last waves there was a statistically significant reduction in the reported prevalence of sharing 'other injection supplies' (cookers, filters or rinse water) in the preceding six months (from an estimated 62% to 40% respectively; P < 0.001) and a concurrent increase in the proportion of those who reported receiving 'other injection supplies' from an HIV prevention provider (from an estimated 18% to 24%, P < 0.004) as well as an increase in reported NSP use (from 5% to 36% respectively; P < 0.001). However, the study found no statistically significant difference in the odds of sharing 'other injection supplies' in the preceding 30 days among those who reported receiving sterile injecting paraphernalia in the preceding 30 days compared to those who did not (AOR 1.11, (0.89-1.39)) (Table 2). Nor was there a statistically significant difference in the sharing of 'other injection supplies' in the preceding 30 days between those who reported NSP use and those who did not (AOR 0.85 (0.68-1.07)).

### Cross-sectional Studies

The only study identified in this review that correlated the use of sterile non- N/S injecting paraphernalia to (self-reported) HCV infection examined a convenience sample of predominantly male, cocaine injecting IDU (n = 275) recruited from NSPs in Montreal, Canada [25]. Almost one quarter of the study population reported using sterile cookers (23%) and filters (23%) and three quarters (75%) using sterile water for half or more of all injecting episodes in the preceding month. Over a third (37%) reported sharing any non-N/S drug preparation equipment (cookers, filters or water) in the 6 months prior to interview. IDU who reported frequent ('half the time or more' *vs*. 'less than half the time') use of sterile cookers, filters and water in the month prior to interview were less likely to report sharing these items of paraphernalia in the preceding 6 months (AORs 0.42 (0.22 - 0.80)), 0.52 (0.28 - 0.97)) and 0.51 (0.29 - 0.90)) respectively). The overall prevalence of self-reported HCV infection was 66%. Individuals reporting frequent, compared to infrequent, use of sterile cookers (AOR 3.92 (1.58 - 9.70)) and water (AOR 2.93 (1.12 - 7.68)), but not filters (AOR not provided), in the preceding month were significantly more likely to be self-reported HCV-negative.

Of the remaining cross-sectional studies, three [26-28] undertook multivariate analyses, although only one appropriately adjusted for potential confounding variables and presented the results [26]. Longshore *et al *examined injecting risk behaviour according to frequency of NSP attendance (once per month or less, two to four times per month and more than four times per month) [26]. Infrequent NSP users (once per month or less) were significantly more likely to report sharing a cooker in the preceding six months than frequent NSP attendees (more than four times per month) (AOR 2.55 (1.05 - 6.17)). However there was no significant difference between infrequent and frequent NSP attendees in self-reported sharing cotton filters in the preceding six months (AOR 1.57 (0.66 - 3.75)), despite the NSP providing this item of paraphernalia. Kipke *et al *compared injecting risk behaviours in the preceding six months in a convenience sample of young IDU described as NSP users and nonusers, although these groups were poorly defined [27]. Following adjustment for age, gender and ethnicity, NSP users were significantly less likely to report 'sharing cotton, cookers or water' in the preceding six months than nonusers (AOR 0.53 (0.28 - 0.99)). Sears *et al *examined injecting risk behaviours in young homeless IDU recruited from two areas in San Francisco, USA, an intervention site at which a peer-led secondary exchange programme was in operation (n = 67) and a comparison site near which two sanctioned primary NSPs and one clandestine NSP were in operation (n = 55) [28]. Participants recruited from the intervention site were less likely to report using someone else's cotton in the 30 days prior to interview than participants recruited from the comparison site (27% *vs*. 49%, p = 0.003). This difference did not, however, persist following adjustment for potential confounding variables (AOR not reported).

Two further cross-sectional studies were reviewed [29,30]. In their multisite study, Heimer *et al *reported a statistically significant difference between the sharing of water for drug preparation (3% *vs*. 9% respectively; p < 0.001) and syringe rinsing (3% *vs*. 9% respectively; p < 0.005) in the 30 days prior to interview between NSP users and nonusers, but no difference in the sharing of cookers (9% *vs*. 12% respectively) or filters (6% *vs*. 8% respectively) between groups (p values not provided) [29]. Adjusted analyses according to NSP use were not presented and it was noted that NSP had recently aggressively begun distributing sterile water; the other items of paraphernalia provided by NSP were not described in detail. Finally, Guydish *et al *described the frequency of NSP use and the mean percentage of syringes obtained from NSP in relation to self-reported sharing of rinse water (a proxy measure for the sharing of cookers, cottons or rinse water) in the preceding 30 days [30]. The authors reported no significant difference in the mean number of NSP visits in the preceding 30 days among participants reporting sharing rinse water compared to those who did not (4.36 *vs*. 3.75 visits to the NSP in the preceding 30 days respectively). However, those who shared rinse water reported obtaining significantly less syringes from an NSP (86%) compared to non-sharers (89%) (P < 0.05).

### Intervention study

Colon *et al *evaluated a non-randomized, uncontrolled pilot study of a 16 week intervention that aimed to reduce contamination of injecting paraphernalia through the promotion of novel drug preparation practices and devices (a water bottle with dropper for preparing drug solution and rinsing syringes, a N/S pre-fitted with a sterile filter and a hand sanitizer for cleaning hands and injection sites) [31]. Two weeks following the end of the intervention, 56% of study participants reported using water from the water bottle in preparing drug solution, 53% using water from the bottle to rinse a syringe, 66% using the hand sanitizer and 34% using the syringe with pre-fitted filter for at least 75% of all injecting episodes in the day prior to interview and 'most of the time' or 'always' in the 7 days prior to interview; levels indicating potential for self-sustaining change in behaviours. There was no statistically significant reduction in the reported sharing of cookers in the week prior to interview between baseline (16%) and follow up visits (6%) (p = 0.453). However a 66% reduction in the likelihood of detecting red blood cells on items of paraphernalia (water containers and cookers) collected from shooting galleries during the intervention, compared to prior to the intervention, was reported.

## Discussion

### Summary of key findings

This review aimed to explore the evidence base around the provision of non- N/S sterile injecting paraphernalia as an intervention in the primary prevention of HCV among IDU. We did not identify any studies that examined the relationship between the supply of injecting paraphernalia other than N/S and biological measures of HCV infection. One study related self-reported HCV infection to the use of sterile cookers, filters and water, finding that those who frequently used sterile cookers and water but not filters were more likely to self-reported negative HCV status than those who infrequently used them [25]. Inferences about temporality cannot be drawn from this study due to its cross-sectional design. The remaining observational studies reported behavioural outcomes. Eight studies presented adjusted odds ratios for the association between exposure to an NSP or SIF and sharing injecting paraphernalia other than N/S [19-22,24,26-28]. Effect size estimates were suggestive of a reduction in the odds of sharing injecting paraphernalia other than N/S associated with exposure to NSP or SIF, but confidence intervals were wide and often included unity. One study found no significant association between the receipt of non- N/S sterile injecting paraphernalia and the sharing of these items [24]. Studies that examined unadjusted temporal trends in the prevalence of sharing non-N/S injecting paraphernalia [20,21,23,24] reported significant reductions over time, usually coinciding with an increase in NSP use. However, the only study to report an adjusted temporal trend found that prevalence rates of sharing injecting paraphernalia other N/S were lower at each time point in non-NSP users compared to NSP users [21]. The authors report a greater decline over time in non-NSP users compared to NSP users although data to support this statement are not provided in the manuscript.

### Limitations of the current literature

The only non-observational study included in this review was a non-randomized, uncontrolled pilot intervention study with few participants and no long-term follow up [31]. Whilst providing encouraging data to support larger scale intervention trials, these results should be interpreted with caution. Of the observational studies, seven employed cross-sectional designs [24-30], which are limited with regards to the causal inferences that can be drawn. It is possible that IDU who engaged in high risk behaviours were also less likely to use NSP or SIF. In studies examining temporal trends in the prevalence of sharing non- N/S injecting paraphernalia [20,21,23,24], changes in observed injecting practices may be attributable to contemporaneous interventions, such as the delivery of harm reduction advice through NSP, SIF or other service providers.

A constraint exists between the aim of this review and the primary aim of the studies we identified. Examining the effect of providing sterile non-N/S paraphernalia to IDU was often a secondary rather than primary aim. This is reflected in the design, analyses and reporting of these studies. Few studies actually measured the provision or uptake of paraphernalia and, with two notable exceptions [24,25], exposure to NSP was invariably adopted as a proxy measure for uptake of the injecting paraphernalia offered by NSP. Three studies measured the frequency [22,26] or probability [19] of NSP (or SIF) use, but several studies used a more crude binary measure of NSP "users and non-users". The distinction between NSP users and non-users was often unclear and in some cases, both individuals designated as NSP users and non-users had access to the sterile paraphernalia under study [20,24,27,28]. For example, in the study by Huo *et al*, clean filters and water were distributed to all participants [20], whilst in the study by Sears and colleagues participants were classified according to the site of recruitment rather than reported NSP use. In the latter study, use of "any NSP" was almost ubiquitous among participants recruited from the intervention site and as high as 80% of the participants recruited from the comparison site [28]. Misclassification of exposure status may have attenuated any observed association between NSP use and injecting risk behaviours.

As has been shown in the studies included in this review, the sharing of individual items of injecting paraphernalia is often highly correlated. Half of all studies selected the sharing of a single or multiple items of injecting paraphernalia to represent all indirect sharing in their analyses [19-21,24,27,30]. Huo *et al *for example investigated the association between NSP use and the sharing of 'other injecting paraphernalia', denoting sharing of either a cooker, filter or rinse water [20]. Thus, it is not possible, in this review, to isolate the impact of paraphernalia provision on the sharing of any specific item of paraphernalia.

All studies used interviewer administered questionnaires or structured interviews to ascertain injecting risk behaviour and may therefore be subject to recall and social desirability bias [19-30]. It is reported that IDU reliably describe injecting risk behaviours [32] however ethnographic studies suggest that IDU may not be aware of episodes of indirect sharing [7]. Differential reporting of sharing practices could result in residual confounding which would affect any reported association.

Only two studies recruited a random sample of IDU [19,22]; the remaining studies adopted a targeted [24,29,31], systematic sampling strategy [21,23,30] or recruited convenience samples [20,25-27] and often offered financial incentive to participants [19,21-23,25-28,30]. All the studies identified were conducted in North America and several examined specific groups of IDU (for example homeless youth [28] or predominantly male, cocaine injectors [25]), in particular settings or contexts (for example SIF [22] or secondary syringe exchange programmes [28]). IDU are not a homogeneous group. Differences in the patterns of sharing of drug injecting equipment, the perceived risks associated with this and use of NSP have been reported according to key sociodemographic such as gender [33]. In addition important differences in structural and organisation components of NSP, the provision of and access to health care and the wider socio-political climate that may limit the generalisability of these findings to other populations.

A number of studies failed to adjust for potential confounders [23,29,30], or adjusted for only socio-demographic characteristics in statistical analyses [27]. It is likely therefore that any effect from these studies will be subject to residual confounding. Effective sample sizes of the included studies ranged from 32 to 1582. The larger studies [19,20,24] were able to estimate effect sizes with more precision, but given that no sample size calculations were presented, it is uncertain whether studies were adequately powered to detect an effect. Additionally, given that injecting behaviours were highly correlated, it is possible that even the largest studies would have had insufficient power to detect an association between an isolated injecting risk behaviour and NSP attendance. Indeed the largest of the cohort studies, Hagan *et al *experienced substantial losses to follow-up and analysed data on only 56% of all participants enrolled at baseline which will have further reduced ability to detect an association [19].

### The wider context

There is limited evidence that HCV transmission occurs through the indirect sharing of injecting paraphernalia [13]. Nevertheless the theoretical risks of HCV transmission through this route have been recognised for over a decade [11] and high rates of sharing other injecting paraphernalia have consistently been reported. Indeed the sharing of cookers, filters and water is often reported to be much more common than the sharing of N/S. Many of the challenges of undertaking research in this area have been highlighted in the methodological limitations of the studies included in this review. Harm reduction interventions such as NSP are complex and as such demonstrating the effectiveness of each active component of the intervention is challenging. In addition, access to sterile injecting paraphernalia other than N/S at NSP or other settings does not necessarily translate into uptake or use of paraphernalia by IDU. Qualitative studies from the UK and elsewhere suggest that many IDU do not fully appreciate the risks associated with sharing non- N/S injecting paraphernalia [33]. Situational (convenience, ease of access) and social factors (knowledge of injecting partners, pooling of resources) [25,33], and levels of satisfaction with, and perceived ease of use of [25], sterile injecting equipment are also important in determining use. A further challenge lies in demonstrating the impact of an intervention on HCV transmission: the prevalence of HCV in IDU populations is generally high, therefore identifying a large cohort of HCV naïve IDU to prospectively study, and ensuring low rates of attrition, can be difficult. However new developments in the serosurveillance of IDU [34,35] offer the potential to examine the impact of interventions on recently acquired HCV infection using a cross-sectional design [34,35].

### Limitations of this review

Given the heterogeneity in study designs, settings, populations and outcomes, we have not been able to present an overall measure of effect in this review. We conducted a thorough search of the literature, including the grey literature, to identify all potentially relevant material. However a number of studies were excluded because a detailed description of the injecting supplies provided by NSP was not available; improved reporting in primary studies may have yielded more evidence. This review examined biological (prevalent and incident HCV infection) outcomes, although this did not yield any studies, and behavioural (injecting risk behaviours) outcomes. It is important to acknowledge that the use of sterile non- N/S injecting paraphernalia may have additional benefits that have not been captured in outcomes considered in this review; for example, the potential to reduce bacterial infections or to attract and engage IDU who may not otherwise be in contact with health services [18]. Policy decisions on the distribution of non-N/S paraphernalia should not therefore be made on the basis of evidence relation to HCV transmission or injecting risk behaviour in isolation.

## Conclusions

Current evidence suggests that attendance at NSP providing sterile non-N/S injecting paraphernalia may be associated with reduced sharing of non-N/S injecting paraphernalia. However, the evidence is limited by the number and quality of the studies. Therefore robust conclusions cannot be drawn in relation to the impact of providing non-N/S paraphernalia on injecting risk behaviours. No studies were found to have examined the impact of providing sterile non-N/S injecting paraphernalia on the transmission of HCV. We have identified a critical gap in the literature. Future studies would benefit from longitudinal designs, accurate measurements of the exposure variable as opposed to the use of proxy variables, adjustment for known and potential confounders and should be adequately powered to detect changes in injecting risk behaviour or incident HCV infection between exposed and unexposed groups. These data are critical to inform future public health policy and guide decision making by service providers and users.

## Competing interests

The authors have no financial competing interests to declare. All authors were members of the 'Prevention Short Life Working Group' for Phase I of the Hepatitis C Action Plan for Scotland. This study was commissioned by this group in order to establish the evidence base from which to inform future action on prevention in Phase II of the Hepatitis C Action Plan for Scotland.

## Authors' contributions

NP, SH, SA, AT, DG devised the research question. MG and NP devised the search strategy, identified and appraised relevant literature. SH acted as third reviewer, assisting in appraisal and interpretation of relevant studies where agreement could not be met. MG drafted the manuscript with critical input from all other authors. All authors read and approved the final manuscript.

## Appendix 1

(Table 3)

**Table 3:** Search Strategy


1	(Hepatitis C or HCV).mp. or *Hepatitis C/
2	transmi$.mp. or *Disease transmission/ or *Virus Transmission/ or *Infection risk/
3	(contaminat$ or cross-contaminat$ or crossinfect$ or cross-infect$).mp. or *Equipment Contamination/
4	seroconver$.mp. or *Seroconversion/
5	(behavio?r modification or risk reduc$).mp. or *Risk reduction/ or *Behavior modification/
6	((inject$ or risk) adj1 (behavio?r or practice$)).mp. or *High risk behavior/ or *Risk taking behavior/ or *Risk taking/ or *Risk-taking/ or *Risk Factor/ or *Risk factors/
7	sharing.mp.
8	(needle exchange$ or syringe exchange$ or harm reduction).mp. or *Harm Reduction/ or *Needle-Exchange Programs/
9	((intravenous or inject$ or substance$ or drug$) adj1 (paraphe?nalia or equipment or ap?aratus)).mp.
10	(spoon$ or cooker$ or filter$ or cotton$ or water or stericup$).ti,ab,tw. indirect.mp.
11	("substance$ use$" or "substance$ abuse$" or "substance$ misus$" or "substance$ depend$" or "substance$ addict$" or "drug$ use$" or "drug$ abuse$" or "drug$ misus$" or "drug$ depend$" or "drug$ addict$").mp. or *Substance Abuse, Intravenous/ or *Substance-Related Disorders/ or *Intravenous Drug Usage/ or *Drug Abuse/ or *Drug addiction/ or *Drug dependency/ or *Substance Abuse, Intravenous/ or *Intravenous Drug Users/ or *Substance Abusers/ or *Substance Dependence/ or *Intravenous Drug Abuse/ or *Drug abuse/ or *Drug dependence/
12	((1 and (or/2-4)) or 5 or 6 or ((or/9-11) and 7)) and 8 and 12
13	Limit to English language, human subjects and year = "1989 - Current"

## Pre-publication history

The pre-publication history for this paper can be accessed here:

http://www.biomedcentral.com/1471-2458/10/721/prepub
